# Effects of improved amino acid balance diet on lysine mammary utilization, whole body protein turnover and muscle protein breakdown on lactating sows

**DOI:** 10.1186/s40104-024-01020-9

**Published:** 2024-05-07

**Authors:** Sai Zhang, Juan C. Marini, Vengai Mavangira, Andrew Claude, Julie Moore, Mahmoud A. Mohammad, Nathalie L. Trottier

**Affiliations:** 1 Department of Animal Science, Michigan State University, East Lansing, 48824 USA; 2 State Key Laboratory of Swine and Poultry Breeding Industry, Key Laboratory of Animal Nutrition and Feed Science in South China, Ministry of Agriculture and Rural Affairs, Guangdong Provincial Key Laboratory of Animal Breeding and Nutrition, Institute of Animal Science, Guangdong Academy of Agricultural Sciences, Guangzhou, 510640 PR China; 3 USDA/ARS Children’s Nutrition Research Center and Pediatric Critical Care Medicine, Department of Pediatrics, Baylor College of Medicine, Houston, TX 77030 USA; 4 Department of Large Animal Clinical Sciences, Michigan State University, East Lansing, MI 48824 USA; 5 Present address: Department of Animal Science, Cornell University, Frank Morrison Hall, 507 Tower Road, Ithaca, NY 14853-4801 USA

**Keywords:** Amino acid, Efficiency, Lactating sows, Protein breakdown, Protein turnover, Reduced protein diet

## Abstract

**Background:**

The study objective was to test the hypothesis that low crude protein (CP) diet with crystalline amino acids (CAA) supplementation improves Lys utilization efficiency for milk production and reduces protein turnover and muscle protein breakdown. Eighteen lactating multiparous Yorkshire sows were allotted to 1 of 2 isocaloric diets (10.80 MJ/kg net energy): control (CON; 19.24% CP) and reduced CP with “optimal” AA profile (OPT; 14.00% CP). Sow body weight and backfat were recorded on d 1 and 21 of lactation and piglets were weighed on d 1, 14, 18, and 21 of lactation. Between d 14 and 18, a subset of 9 sows (CON = 4, OPT = 5) was infused with a mixed solution of 3-[methyl-^2^H_3_]histidine (bolus injection) and [^13^C]bicarbonate (priming dose) first, then a constant 2-h [^13^C]bicarbonate infusion followed by a 6-h primed constant [1-^13^C]lysine infusion. Serial blood and milk sampling were performed to determine plasma and milk Lys enrichment, Lys oxidation rate, whole body protein turnover, and muscle protein breakdown.

**Results:**

Over the 21-d lactation period, compared to CON, sows fed OPT had greater litter growth rate (*P <* 0.05). Compared to CON, sows fed OPT had greater efficiency of Lys (*P <* 0.05), Lys mammary flux (*P <* 0.01) and whole-body protein turnover efficiency (*P <* 0.05). Compared to CON, sows fed OPT tended to have lower whole body protein breakdown rate (*P* = 0.069). Muscle protein breakdown rate did not differ between OPT and CON (*P* = 0.197).

**Conclusion:**

Feeding an improved AA balance diet increased efficiency of Lys and reduced whole-body protein turnover and protein breakdown. These results imply that the lower maternal N retention observed in lactating sows fed improved AA balance diets in previous studies may be a result of greater partitioning of AA towards milk rather than greater body protein breakdown.

## Background

The increasing availability of crystalline amino acid (CAA) at competitive costs relative to protein ingredients allows for reduction of excessive dietary nitrogen (N) and improving AA balance [1]. Several studies have shown that improving dietary AA balance in lactating sows leads to greater milk casein yield [2, 3] and utilization efficiency of N and essential amino acid (EAA) [4, 5] while dramatically mitigating N losses and ammonia emissions to the environment [2]. Lysine efficiency values previously reported [4–6] were estimated based on lean mass change during lactation. This approach yielded similar efficiency estimates for Val based on isotopic method [7]. Lysine utilization efficiency values in lactation using an isotopic approach have not been reported.

The increased apparent AA efficiency may be at the expense of sow body weight (BW) loss and reduced maternal N retention whereby partitioning of dietary AA and energy towards the mammary glands appears to be favoured [3, 5, 8]. Preserving maternal N pool during the lactation period is important since maternal body protein and lipid loss can affect subsequent production performance. Loss in performances may include delayed estrus [9], reduction of piglet birth weight and litter uniformity [10, 11], and prolonged interval from weaning to successful pregnancy [12], thus compromising the overall life span production efficiency. It is unknown whether the reduced maternal N retention previously reported [3, 5] in sows fed an improved AA balance diet was a result of greater maternal body protein breakdown. In addition, whole body and muscle protein breakdown rates in lactating sows are unknown and such values are critical to assess at a mechanistic level the impact of improved dietary AA balance on body protein dynamic. Isotope technique allows for better mechanistic understanding of protein dynamics, including protein turnover rate, AA flux and muscle protein breakdown in humans [13] and animals [14].

We hypothesized that low CP diet with improved AA balance would increase milk yield through improving efficiency of Lys for milk and increasing maternal body and muscle protein breakdown. The objectives were to (1) measure whole body protein dynamics and (2) estimate Lys utilization efficiency for milk synthesis.

## Materials and methods

### Dietary treatments

The NRC model [6] was used to estimate requirements for AA, net energy (NE), calcium (Ca) and phosphorus (P). The requirements were predicted based on the following parameters: sow BW of 210 kg after parturition, parity number of 2 and above, average daily intake of 6 kg/d, litter size of 10, piglet average daily gain (ADG) of 280 g/d over a 21-d lactation period, and an ambient temperature of 20 °C. The model predicted a minimum sow BW loss of 7.5 kg and the protein to lipid ratio in the model was adjusted to the minimum allowable value of near zero. The model predicted SID Lys requirement of 0.90% and NE requirement of 2,580 kcal/kg.

A control diet (CON) was formulated using corn and soybean meal as the only sources of Lys to meet the SID Lys requirement (0.90%) and consequently contained 19.24% CP and a SID Val concentration 0.77% which was near the NRC (2012) requirement of 0.79%. All other EAA SID contents were in excess relative to NRC (2012). The SID AA values of feed ingredients were referred to NRC (2012). A second diet was formulated to improve AA balance [5], and referred to as optimal diet (OPT) throughout the manuscript. Fermentable fiber was high in CON due to high content of soybean meal with 24.88% fermentable fiber [6]. Thus, the same fiber source (soy hulls) was supplemented in OPT, and levels of fermentable fiber were consistent between CON and OPT. Ingredients and calculated nutrient composition of CON and OPT diets are presented in Table 1. Analyzed total (hydrolysate) and free AA concentrations are presented in Table 2, in order to verify the precision of diet formulation. The analyzed N concentration corresponded to a CP% of 18.44 compared to a calculated value of 19.24% CP. Therefore, the analyzed CP concentration value is used in the heading for the remainder of tables.

**Table 1:** Ingredient composition and nutrient content of experimental diets (as-fed)

Item	Control (CON)	Optimal (OPT)
Ingredient composition, %		
Corn (yellow dent)	59.17	61.45
Soybean meal (48 % CP)	30.00	14.00
Soy hulls	0	10.57
Sugar food product^1^	5.00	5.00
Beef tallow	3.35	5.02
l-Lys·HCl	0	0.47
l-Val	0	0.29
l-Thr	0	0.20
l-Phe	0	0.13
dl-Met	0	0.11
l-Ile	0	0.08
l-His	0	0.07
l-Trp	0	0.05
l-Leu	0	0
Limestone	1.18	0.93
Dicalcium phosphate	0.45	0.78
Sodium chloride	0.50	0.50
Vitamin and mineral premix^2^	0.25	0.25
Titanium dioxide	0.10	0.10
Total	100.00	100.00
Calculated nutrient concentration^3^		
NE, kcal/kg	2,580	2,580
CP, %	19.24	14.00
Fermentable fiber, %	11.58	11.58
SID^4^ AA, %		
Arg	1.17	0.71
His	0.47	0.37
Ile	0.71	0.52
Leu	1.47	1.03
Lys	0.90	0.90
Met^5^	0.27	0.30
Met + Cys	0.54	0.49
Phe	0.84	0.67
Phe + Tyr	1.38	1.03
Thr	0.61	0.58
Trp	0.21	0.17
Val	0.77	0.79
Total Ca, %^6^	0.65	0.65
STTD P, %^6^	0.23	0.23

^1^Supplied per kg: NE 2,842 kcal; fermentable fiber 0.05 %; CP 1.00 % (International Ingredient Corporation, St. Louis, MO, USA)

^2^Sow micro 5 and Se-yeast PIDX15 (Provimi North America, Inc., Brookville, OH, USA)

^3^Based on nutrient concentrations in feed ingredients according to NRC [6]

^4^*SID* = Standardized ileal digestible [6]

^5^Met concentration in OPT is higher than CON because Met was added to meet Cys requirement (Met + Cys)

^6^Concentrations of Ca and P were based on phytase activity from the premix

**Table 2:** Analyzed and calculated concentration of nitrogen (N), total and free essential amino acids in control (CON) and optimal (OPT) diets (as-fed)

Item	CON		OPT	
	Analyzed^1^	Calculated^2^	Analyzed^1^	Calculated^2^
Total, %				
N	2.95	3.08	2.24	2.24
Arg	1.18	1.26	0.70	0.78
His	0.51	0.53	0.40	0.43
Ile	0.84	0.81	0.60	0.60
Leu	1.60	1.67	1.10	1.19
Lys	1.06	1.04	1.03	1.01
Met	0.26	0.31	0.26	0.33
Met + Cys	0.55	0.63	0.47	0.57
Phe	0.95	0.96	0.73	0.76
Phe + Tyr	1.52	1.59	1.13	1.20
Thr	0.69	0.73	0.61	0.68
Trp^3^	0.22	0.23	0.17	0.19
Val	0.91	0.90	0.87	0.89
Free AA, %				
Arg	0.05	0.00	0.03	0.00
His	0.00	0.00	0.07	0.07
Ile	0.01	0.00	0.08	0.08
Leu	0.01	0.00	0.01	0.00
Lys	0.02	0.00	0.41	0.37
Met	0.00	0.00	0.10	0.11
Met + Cys	0.00	0.00	0.10	0.11
Phe	0.00	0.00	0.13	0.13
Phe + Tyr	0.01	0.00	0.15	0.13
Thr	0.02	0.00	0.21	0.20
Trp^3^	-	0.00	-	0.05
Val	0.01	0.00	0.27	0.29

^1^Analyzed values represent average across 3 blocks (feed mixes)

^2^Calculated values for the total AA are based on the AA concentration in feed ingredients according to NRC [6], and calculated values for the free AA correspond to the dietary inclusion rate in crystalline form

^3^Analysis of free Trp was not performed

### Animals and feeding

The study was conducted at the Michigan State University Swine Teaching and Research Center. A total of 18 purebred multiparous (parity 2+) Yorkshire sows were moved to conventional farrowing crates between d 105 and 107 of gestation, grouped by parity, and randomly assigned to 1 of 2 dietary treatments within parity groups (CON, *n* = 9; OPT, *n* = 9). The study was conducted over 3 blocks of time, with 6, 6, and 5 sows in each block, respectively. One sow in CON from block 3 was removed due to poor feed intake that was deemed unrelated to the dietary treatments. Sows were adapted to the experimental diets (2.2 kg/d) 4 to 6 d before the expected farrowing date. Following farrowing, sows feed allowance progressively increased from 1.88 kg/d on d 1 to 7.44 kg/d at d 21, according to the NRC model [6], with a targeted ADFI of 6.0 kg/d over the 21-d lactation period. Feed was provided daily in 3 equal meals (0700, 1300, and 1900) with feed intake and refusal recorded daily before the morning meal. On infusion days (between d 14 and 18), the 0700 and 1300 meals were divided into 6 aliquots fed every 2 h from 0700 to 1700. Water was freely accessible to sows and piglets. Litters were aimed to be standardized to 11 piglets within the first 24 h after farrowing with the objective of weaning 10 piglets per sow. Injection of iron and surgical castration of male were conducted on d 1 and 7, respectively, according to the institutional research farm protocol. No creep feed was supplied to the piglets. Body weight and backfat thickness [5] of sows were recorded on d 1 and 21, and litter weights were recorded on d 1, 14, 18 and 21. Milk yield was estimated for peak lactation (between d 14 and 18) [5]. Prediction equation for milk yield during peak lactation is as follows [15]: $\mathrm{Daily}\;\mathrm{milk}\;\mathrm{yield}\;(\text{g}/\text{d})=\mathrm{littersize}\times(582+1.168\times\text{ADG}+0.00425\times\text{ADG}^2)$

### Bilateral ear vein catheterization

A subset of 10 sows (5 sows per treatment) was used for the catheterization and infusion protocol. An ear vein catheter was placed in each ear, with one ear serving as the infusion line and the other as the sampling line. For the length of the catheterization procedure, piglets were removed and transferred to an empty adjacent stall with a heat lamp. The sows were restrained with a rope snare and remained in their farrowing stall where sedation was induced. For sedation, Telazol was reconstituted with 2.5 mL of 100 mg/mL ketamine and 2.5 mL of 100 mg/mL xylazine to a volume of 5 mL. This sedative mixture was administered i.m. in the brachiocephalicus muscle approximately 6 cm caudal to the ear, at a dosage of 0.1 mL/4.537 kg body weight. Sows were carefully assisted to facilitate laying in ventral recumbence. Sedation lasted for 45 to 60 min. The depth of anesthesia was monitored by the degree of muscle relaxation and respiration rate (i.e., 10 to 25 breaths/min).

The entire dorsal surface of both ears was prepared for aseptic placement of the ear vein catheters. The skin was scrubbed gently with 10% betadine solution followed with 70% isopropyl alcohol. The hair covering the skin area caudal to the ear and dorsal to the neck was clipped using a professional clipper to ensure a good adhesion of veterinary adhesive tape to the skin (described below).

A pre-cut 61-cm, round tip, medical grade microbore intravascular tubing (1.65 mm o.d., 1.02 mm i.d.) with hydromer coating (Access Technology Corp., Skokie, IL, USA) was prefilled at the time of catheterization with heparinized saline (30 IU/mL) before insertion. A hand tourniquet was applied at the base of the ear to distend the medial and lateral branches of the auricular vein. Either vein was used for catheterization. A short-term stylet catheter (14G, 5.08 cm, Safety IV catheter; B. Braun Melsungen AG, Germany) was inserted into the vein with the needle bevel facing up. Upon appearance of blood, the vein was gently occluded, and the needle rotated 180° to angle the bevel facing down. While holding the needle in place, the stylet catheter was gently pushed into the vein through the needle. Once the stylet was in place, the needle was removed, and the intravascular tubing was inserted through the stylet and pushed for approximately 30 cm caudally to reach the external jugular vein, and the catheter verified for patency at this point. Small sections of tape (5.1 cm wide, ZONAS® porous tape, Johnson & Johnson Consumer Companies, Inc., Skillman, NJ, USA) were affixed to the remaining section of intravascular tubing and sutured to the skin to secure the tubing in place. The stylet catheter was also sutured (Monocryl, CP-1, 36 mm, 1/2c; Ethicon Inc., USA) to the skin at the point of entry. Gauze was placed over each sutured sites and held in position by wrapping the ear with elastic adhesive tape. A connector was used to join the intravascular tubing to a long tubing extension (approximately 120 cm). A blunt-end needle adapter with an adaptor injection cap and a male luer lock was placed onto the distal end of the tubing extension. The same vein catheterization procedure was done on the other ear. A final layer of elastic adhesive tape (7.5 cm wide, 3M veterinary adhesive tape) was used to wrap each ear into a gently folded cone shape and to affix extension tubing directly onto the clipped skin surface. The extension tubing ran from the ears to the dorsal region of the neck, caudally to the ears and cranial to the shoulders and the free end (approximately 100 cm) rolled up and placed in a handmade denim protective pouch mounted on 4.0-cm thick foam material. The pouch was kept in place by gluing the foam directly onto the skin with Livestock ID Tag Cement (W.J. Ruscoe Company, Akron, OH, USA). Catheters were verified for patency once more and the lines were filled with sterile saline, coiled, and placed in the pouch until used for infusion and blood sampling. The entire procedure was done following sterile techniques and lasted 45 to 90 min per sow. As soon as sows were able to stand, 15-cm wide elastic bandage (Novation^®^, Hartmann USA, Inc., Rock Hill, SC, USA) was wrapped over the pouch and around the neck and thorax in at least 3 layers in the shape of a life vest (crisscross) to protect the pouch. Thereafter, the catheters were verified for patency and flushed with sterilized heparinized saline (30 IU/mL) twice per day.

**Fig. 1:**
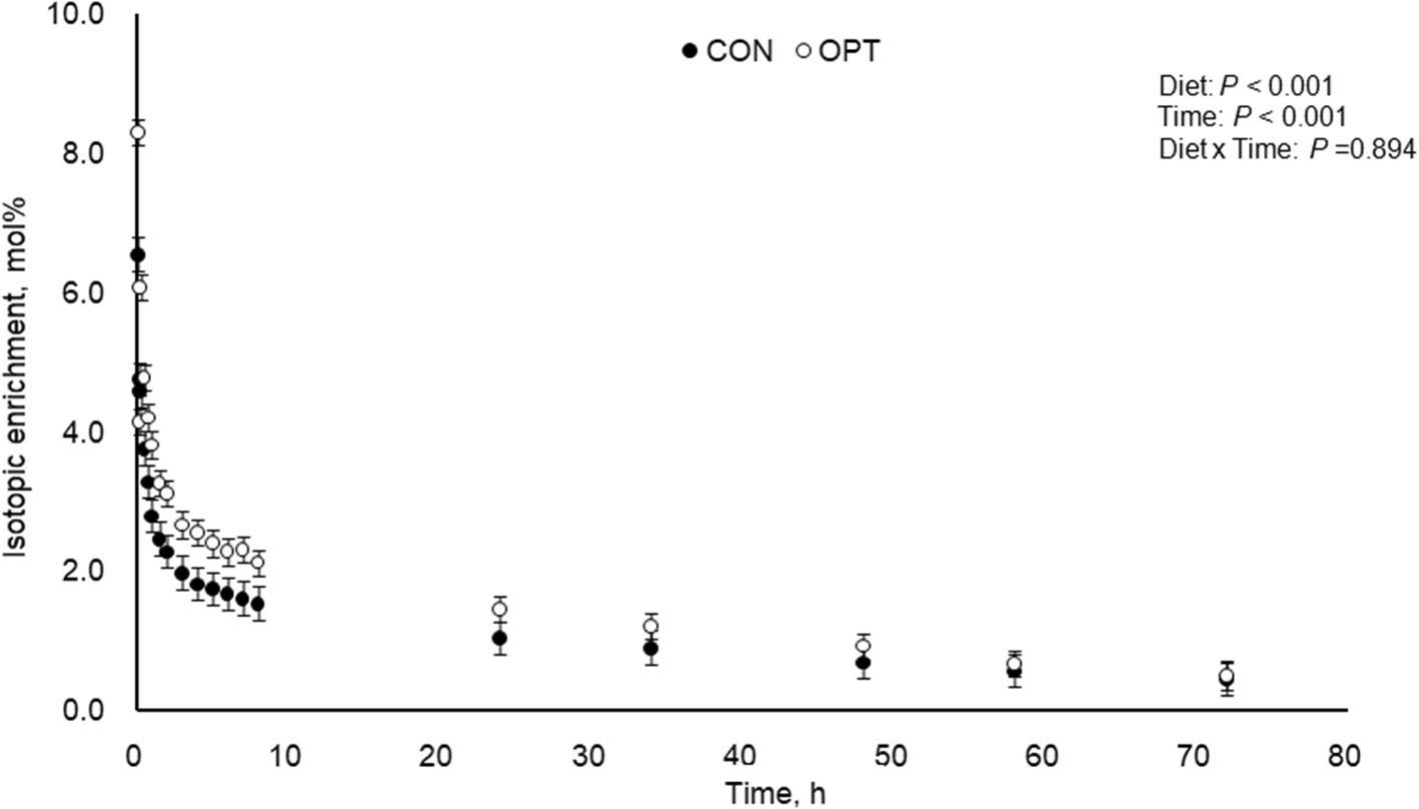
Plasma isotopic enrichment of 3-[methyl-^2^H_3_]histidine following 3-[methyl-^2^H_3_]histidine bolus infusion during peak lactation (between d 14 and 18) for sows fed control (CON; 18.4% CP; *n* = 4) and optimal (OPT; 14.0% CP; *n* = 4) diets. Plasma isotopic enrichment of 3-[methyl-^2^H_3_]histidine differed between diets (*P* < 0.001) and time points (*P* < 0.001), with no interaction between diet and time (*P* = 0.894). Standard error of the mean (SEM) = 0.214

Catheters were removed after all infusions and blood sampling were completed (blood sampling lasted for 3 d for 3MH; Fig. 1). The elastic bandaging was removed, and the elastic adhesive tape was carefully pulled to expose the sutures. The sutures were cut with small surgical scissors, the catheters were gently pulled out of the ear veins, and pressure was applied over the insertion sites to accelerate coagulation. The remaining adhesive tape was then carefully removed, and the pouch was freed from the foam which remained on the sow. Rectal temperature was recorded from the day of catheterization and for 3 d following removal of catheters.

### Preparation of isotope solutions

Tracers were weighed, dissolved in saline and the solution sterilized by filtration through Millipore Steriflip filters (0.22 μm). For each sow, the following stock solutions were prepared: 3-[methyl-^2^H_3_]histidine (183 μmol in 20 mL saline for bolus injection), [^13^C]bicarbonate (368 μmol in 20 mL saline for prime and 736 μmol in 30 mL saline for 2-h infusion), and [1-^13^C]lysine (1.28 mmol in 30 mL saline for prime and 9.00 mmol in 60 mL saline for 6-h infusion). The bolus dose of 3-[methyl-^2^H_3_]histidine (3MH) was calculated based on 20% pool size of 3MH in sows [16, 17]. The infusion rate of [1-^13^C]lysine was calculated based on average flux of lysine (25 mmol/h) in lactating sows [7] with the aim of 2% enrichment. The priming dose of [1-^13^C]lysine was aiming for 1.5 mmol (1 h of infusion), and 1.28 mmol was the actual amount according to weight balance.

**Fig. 2:**
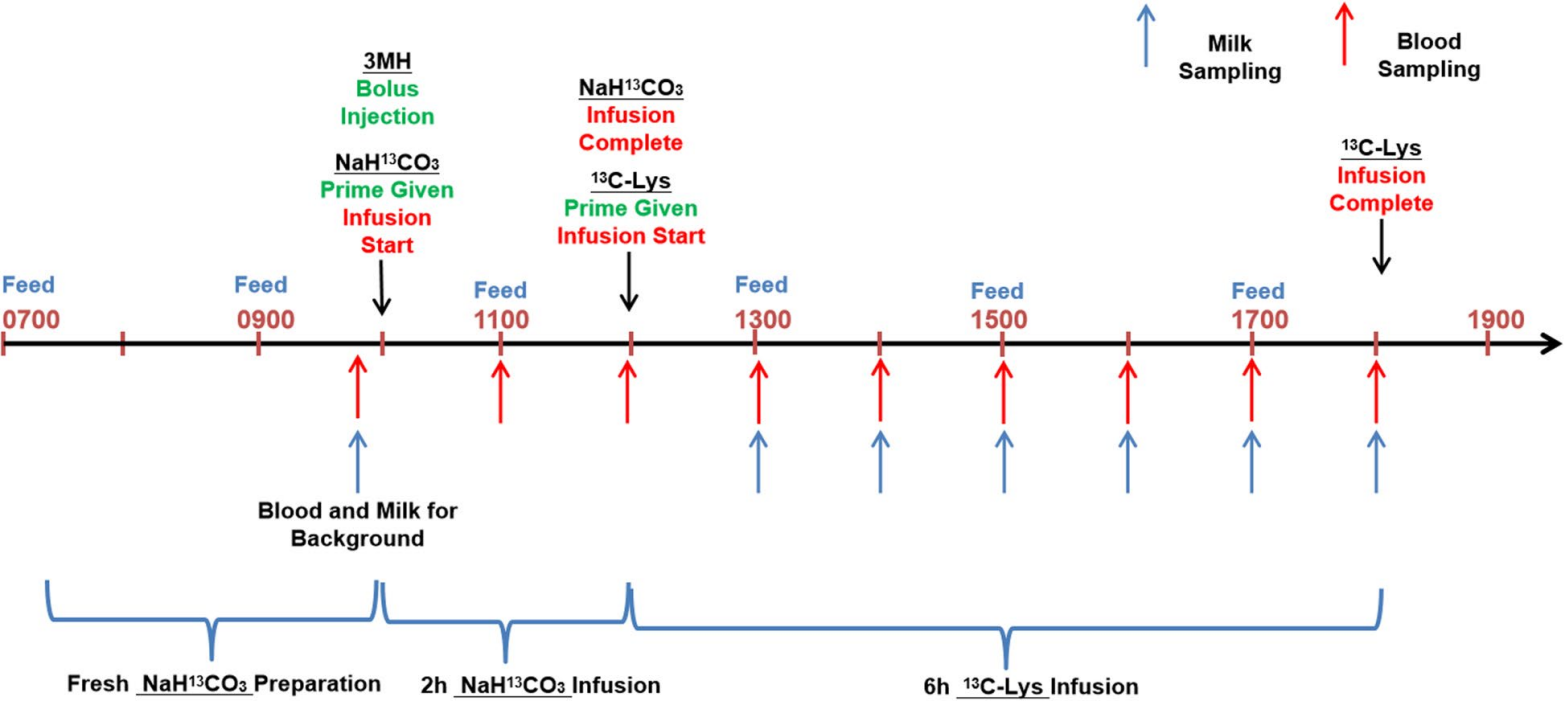
Timeline of isotope infusion and sampling (infusion day was within d 18–21)

The solution of [^13^C]bicarbonate was freshly prepared to minimize loss of ^13^CO_2_. Specifically, [^13^C]bicarbonate was weighed and dissolved in 20-mL 3-[methyl-^2^H_3_]histidine solution in the morning of infusion day (Fig. 2). The 3-[methyl-^2^H_3_]histidine (3MH) was used to estimate muscle protein breakdown, and the [^13^C]bicarbonate was used to prime the CO_2_ pool to accelerate the estimation of lysine oxidation rate. The primed-constant infusion of [1-^13^C]lysine was used to estimate lysine utilization by the mammary gland and the lysine flux in the whole body.

### Infusion protocol

The timeline for infusion is presented in Fig. 2. Actual infusion day varied between d 14 and 18 due to real time patency of catheter. For lysine balance (Table 3) and body protein kinetics (Table 6), the actual infusion days were 17.0 ± 1.0 for CON and 17.0 ± 1.4 for OPT. For 3MH kinetics, the actual infusion days were 17.3 ± 1.0 for CON and 16.8 ± 1.5 for OPT. Pumps (Genie Touch^TM^, Kent Scientific Corp, Torrington, CT, USA) and syringes were placed on a large and stable plastic board laid above the farrowing stall. Following the priming dose, the infusion line was immediately attached to the syringe mounted to the pump to begin the constant infusion. The sampling line was coiled and stored in the pouch until used for blood sampling.

**Table 3:** Lysine balance of sows fed Control (CON; 18.44% CP) and Optimal (OPT; 14.00% CP + CAA) diets during peak lactation (d 14 to 18)^1^

Item	Diet		SEM^2^	*P*-value
	CON	OPT		
No. of sows	3	5		
SID Lys intake, g/d	85.54	87.19	1.30	0.418
Lys oxidation, g/d	15.32	18.25	2.58	0.470
Lys flux, g/d	156.40	131.00	7.44	0.073
Lys from protein breakdown, g/d	70.87	43.79	7.74	0.069
Lys for protein synthesis, g/d	141.08	112.74	9.56	0.109
Lys utilization efficiency^3^, %	50.06	61.78	2.76	0.048

^1^Data are least squares means

^2^Maximum value of the standard error of the means

^3^ $$\text{Efficiency of lysine}=\frac{\text{Lys for protein synthesis }({\text{g}}/{\text{d}})-\text{Lys for protein breakdown }({\text{g}}/{\text{d}})}{\text{Lys flux }\left({\text{g}}/{\text{d}}\right)-\text{Lys oxidation }({\text{g}}/{\text{d}})}$$

The mixed 20 mL saline solution containing 3-[methyl-^2^H_3_]histidine (183 μmol) and [^13^C]bicarbonate (368 μmol) was given through the infusion line as a bolus injection. The [^13^C]bicarbonate in this infusate was used as a priming dose. After bolus injection, a constant 2-h infusion of [^13^C]bicarbonate (368 μmol/h) began. The 2-h [^13^C]bicarbonate infusion was followed by a 6-h primed constant [1-^13^C]lysine infusion (1.50 mmol/h) (Fig. 2).

### Blood sampling

The timeline for blood sampling is presented in Fig. 2. For analysis of plasma 3-[methyl-^2^H_3_]histidine concentrations and estimation of muscle protein breakdown rate, blood samples were collected through the sampling line at 0 (immediately after termination of the bolus infusion), 5, 10, 15, 30 and 45 min and 1, 2, 3, 4, 5, 6, 7, 8, 24, 34, 48, 58 and 72 h post bolus infusion. Blood samples (0.5 mL) were transferred into 500-μL BD microtainer tubes (K_2_EDTA) and centrifuged (1,500 × *g* at 4 °C for 5 min). The plasma was extracted and stored in 1.5-mL microcentrifuge tubes at −20 °C until analysis.

For analysis of plasma [1-^13^C]lysine concentrations and estimation of whole-body Lys flux, blood samples (0.5 mL) were collected prior to infusion for background enrichment and at 1, 2, 3, 4, 5 and 6 h from the start of [1-^13^C]lysine infusion (Fig. 2).

For analysis of blood CO_2_ concentrations, blood samples (2 mL) were collected prior to [^13^C]bicarbonate-prime infusion for background, and at 1, 2, 3, 4, 5, 6, 7 and 8 h following the prime infusion. Blood samples were injected into evacuated vacutainer tubes (Becton Dickinson, Plymouth, UK) previously prepared with 2 mL of phosphoric acid, immediately mixed, and cooled to room temperature. The CO_2_ was then transferred from evacuated vacutainers to Exetainer tubes (Labco Breath Tube, UK) by using pure nitrogen gas as medium until analysis.

### Milk sampling protocol

The timeline for milk sampling is presented in Fig. 2. Milk samples were taken between d 14 and 18 during the infusion protocol. Milk was sampled before infusion for background enrichment, and at 1, 2, 3, 4, 5 and 6 h of primed constant infusion of Lys.

For each milk sampling period, piglets were separated from the sows for 1 h in an empty adjacent farrowing crate with no access to water, and sows were administered 1 mL of oxytocin (20 IU/mL oxytocin, sodium chloride 0.9% w/v, and chlorobutanol 0.5% w/v, VetTek, Blue Springs, MO, USA) through the sampling catheter immediately after blood sampling. The catheter was rinsed with 2 mL of saline solution to ensure oxytocin reached the blood circulation. A total of 30-mL milk was manually collected across all glands and stored in 2 separate 15-mL tubes (polypropylene centrifuge tubes with screw cap, Denville Scientific, Swedesboro, NJ, USA). Piglets were immediately returned to sows to complete nursing and empty the mammary glands. Piglets were removed after nursing and kept separate from the sow until the next milk sampling time, 1 h later.

### Isotope analysis

Plasma and milk [1-^13^C]lysine and 3-[methyl-^2^H_3_]histidine (after acid hydrolysis) were determined as their dansyl derivatives by HESI LC-MS as previously described [18]. The following *m/z* transitions were monitored: 613→379 and 614→380 for [1-^13^C]lysine and 403→124 and 406→127 for 3-[methyl-^2^H_3_]histidine. Determination of blood ^13^CO_2_ enrichment was performed by IRMS (Delta+XL IRMS coupled with GasBench-II peripheral device, Thermo-Quest Finnigan, Bremen, Germany) as previously described [19].

### Nutrient analysis

Feed samples were analyzed for gross energy (GE) by bomb calorimetry (Parr Instrument Inc., Moline, IL, USA). Dry matter and N in feed samples were analyzed as previously described [5]. Dietary AA analysis [AOAC Official Method 982.30 E (a,b,c), 45.3.05, 2006] was performed by the Agricultural Experiment Station Chemical Laboratories (University of Missouri-Columbia, Columbia, MO, USA) as outlined in previous reports [5].

### Calculations

The following assumptions were made during calculation:

1. Priming dose of isotope was assumed to mix with pool instantly.
2. The appearance of unlabeled bicarbonate was constant during the time of primed-constant infusion of bicarbonate (2 h) and that of [1-^13^C]lysine (6 h).
3. [1-^13^C]lysine cannot be synthesized once 1-carbon was lost to CO_2_, thus rate of lysine decarboxylation represented rate of lysine breakdown.
4. Kinetics of plasma lysine was an indicator of kinetics of whole body protein.
5. The indicator AA (lysine) was assumed to be oxidised for maintenance or incorporated into milk protein without other metabolic pathway.

#### Lysine oxidation

The enrichment of CO_2_ during the period of primed-constant infusion of [^13^C]bicarbonate was calculated as follows (Eq. 1):

1 $${\text{E}}_{{\text{CO}}_2}\ (\%)=\frac{\mathrm{Infusion}\;{\mathrm{rate}}_{\text{H}^{13}\text{CO}_3^-}\ (\upmu\mathrm m\mathrm o\mathrm l/\text{h})}{{\text{Ra}}_{\text{HCO}_3^-}\ (\upmu\mathrm m\mathrm o\mathrm l/\text{h})}$$

Where “infusion ${\text{rate}}_{\text{H}^{13}\text{CO}_3^-}$” represents the infusion rate (368 μmol/h) of [^13^C]bicarbonate, and “$\text{Ra}_{\text{HCO}_{3}^-}$” represents the rate of appearance of unlabeled bicarbonate (baseline) in the body.

The enrichment of CO_2_ during the period of primed-constant infusion of [1-^13^C]lysine was calculated as follows (Eq. 2):

2 $${{\text{E}}}_{{{\text{CO}}}_{2}}^{\mathrm{^{\prime}}}\ (\mathrm{\%})=\frac{{{\text{Ra}}}_{{{\text{H}}^{13}{\text{CO}}}_{3}^{-}}\ (\upmu \mathrm{mol}/{\text{h}})}{{{\text{Ra}}}_{{{\text{HCO}}}_{3}^{-}}\ (\upmu \mathrm{mol}/{\text{h}})}$$

Where “${\text{Ra}}_{\text{H}^{13}\text{CO}_3^-}$” represents the rate of appearance of labeled bicarbonate from [1-^13^C]lysine oxidation, and “$\text{Ra}_{\text{HCO}_{3}^-}$” represents the rate of appearance of unlabeled bicarbonate (baseline) in the body as in Eq. 1.

The enrichment of lysine during the period of primed-constant infusion of [1-^13^C]lysine was calculated as follows (Eq. 3):

3 $${\text{E}}_\text{Lys}\ (\%)=\frac{\mathrm{Infusion}\;{\mathrm{rate}}_{\left[1-^{13}\text{C}\right]\text{Lys}}\ (\text{mmol}/\text{h})}{{\text{Ra}}_\text{Lys}\ (\text{mmol}/\text{h})}=\frac{{\text{Ra}}_{{\text{H}^{13}\text{CO}}_3^-}\ (\upmu\mathrm m\mathrm o\mathrm l/\text{h})}{{\text{Ra}}_{{\text{H}^{13}\text{CO}}_3^-\mathrm{from}\;\mathrm{Lys}\;\mathrm{oxidation}}\ (\upmu\mathrm m\mathrm o\mathrm l/\text{h})}$$

Where Ra_Lys_ represents the rate of appearance of unlabeled lysine in the body.

Lysine oxidation was estimated as follows (Eq. 4):

4 $$\text{Lys oxidation}\ (\upmu\mathrm{mol}/{\text{h}})={{\text{Ra}}}_{{{\text{H}}^{13}{\text{CO}}}_{3}^{-}\text{ from Lys oxidation}}\ (\upmu\mathrm{mol}/{\text{h}})=\frac{{{\text{E}}}_{{{\text{CO}}}_{2}}^{\mathrm{^{\prime}}}\ (\mathrm{\%})}{{{\text{E}}}_{{\text{Lys}}}\ (\mathrm{\%})}\times \frac{{\text{Infusion rate}}_{{\text{H}}^{13}{{\text{CO}}}_{3}^{-}}\ (\upmu\mathrm{mol}/{\text{h}})}{{{\text{E}}}_{{{\text{CO}}}_{2}}\ (\mathrm{\%})}$$

#### Whole body protein breakdown and synthesis

**Fig. 3:**
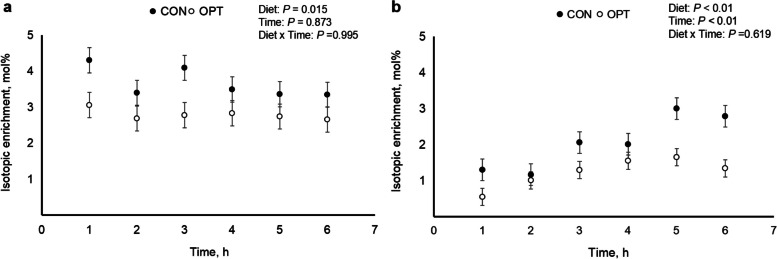
Isotopic enrichment of [1-^13^C]lysine in plasma (panel **a**) and milk (panel **b**) over 6 h during peak lactation (d 14 to 18) for sows fed control (CON; 18.4% CP; *n* = 3) and optimal (OPT; 14.0% CP; *n* = 5) diets

Whole body protein breakdown (PB) and synthesis (PS) were mirrored by Lys dynamics (Table 3 and Fig. 3).

The PB and PS were calculated as follows (Eqs. 5 and 6):

5 $$\text{Lys for PB}\ \left({\text{mmol}}/{\text{h}}\right)={{\text{Ra}}}_{{\text{Lys}}}\ \left({\text{mmol}}/{\text{h}}\right)-\text{Lys intake}\ \left({\text{mmol}}/{\text{h}}\right)\times \mathrm{SID}\ \left(\mathrm{\%}\right)=\frac{{\text{Infusion rate}}_{\left[1-^{13}{\text{C}}\right]{\text{Lys}}}\ \left({\text{mmol}}/{\text{h}}\right)}{{{\text{E}}}_{{\text{Lys}}}\ \left(\mathrm{\%}\right)}-\text{Lys intake}\ \left({\text{mmol}}/{\text{h}}\right)\times \mathrm{SID}\ \left(\mathrm{\%}\right)$$

6 $$\text{Lys for PS}\ \left({\text{mmol}}/{\text{h}}\right)={{\text{Ra}}}_{{\text{Lys}}}\ \left({\text{mmol}}/{\text{h}}\right)-\text{Total Lys oxidation}\ ({\text{mmol}}/{\text{h}})=\frac{{\text{Infusion rate}}_{\left[1-^{13}{\text{C}}\right]{\text{Lys}}}\ \left({\text{mmol}}/{\text{h}}\right)}{{{\text{E}}}_{{\text{Lys}}}\ \left(\mathrm{\%}\right)}-\text{Total Lys oxidation}\ ({\text{mmol}}/{\text{h}})$$

7 $$\text{Protein breakdown or synthesis}\ ({\text{g}}/{\text{d}})=\frac{\text{Lys for PB or PS }({\text{mmol}}/{\text{h}})\times 146.19/(1000\times 24\ {\text{h}})}{6.74\mathrm{\%}}$$

Where 146.19 g/mol is the molar weight of isotopic Lys, 6.74% is the average weight percentage of Lys in the sow’s body protein [6].

8 $$\text{Whole body net protein synthesis}\ ({\text{g}}/{\text{d}})=\text{Protein synthesis}\ ({\text{g}}/{\text{d}})-\text{protein breakdown}\ ({\text{g}}/{\text{d}})$$

9 $$\text{Milk protein yield }({\text{g}}/{\text{d}})=\text{Milk protein concentration}\ (\mathrm{\%})\times \text{estimated milk yield }({\text{g}}/{\text{d}})$$

The average milk protein concentration of 5.16% was used [6]. Milk yield was estimated according to Theil et al. [15].

#### Muscle protein breakdown

Data was expressed as tracer to tracee ratio (TTR). Multiexponential models were fitted to the data (Eq. 10 and Fig. 1). Residual inspection and pseudo-R^2^ were used to determine the most parsimonious model that best fitted the data from each individual sow. Area under the curve (AUC; TTR•h) was calculated using the parameters from the multiexponential equation (Eq. 11 and Table 4) and 3MH rate of appearance (Ra; μmol/kg/h) was calculated by dividing the dose administered (μmol/kg) by AUC (Eq. 12 and Table 4). Half-life (h) was determined using the rate constant corresponding to the tail of the curve (Eq. 13, Table 4, and Fig. 1).

10 $${{\text{TTR}}}_{{\text{t}}}=\sum\nolimits_{{\text{i}}=1}^{{\text{n}}}{{\text{A}}}_{{\text{i}}}\times {{\text{e}}}^{{-{\text{k}}}_{{\text{i}}}\times {\text{t}}}$$

11 $${\text{AUC}}=\sum\nolimits_{{\text{i}}=1}^{{\text{n}}}\frac{{{\text{A}}}_{{\text{i}}}}{{{\text{k}}}_{{\text{i}}}}$$

12 $${{\text{Ra}}}_{3{\text{MH}}}=\frac{\mathrm{Bolus\ dose}}{{\text{AUC}}}$$

13 $$\mathrm{Half\ life}=\frac{{\text{Ln}}(2)}{{{\text{k}}}_{{\text{n}}}}$$

Muscle protein breakdown rate (%/d) was calculated as follows (Eq. 14):

14 $$\text{Muscle protein breakdown rate }(\mathrm{\%}/{\text{d}})=\frac{{{\text{Ra}}}_{3{\text{MH}}}\ (\mathrm{\upmu mol}/{\text{d}})\times 100}{\text{Total protein bound }3\text{MH pool }(\mathrm{\upmu mol})}$$

Where total protein bound 3MH pool = muscle protein mass (g) × 3.8742 μmol 3MH/g protein and muscle protein mass = 8.21% × sow BW (kg) [20].

Muscle protein breakdown (g/d), was calculated as follows (Eq. 15):

15 $$\text{Muscle protein breakdown }({\text{g}}/{\text{d}})=\frac{{{\text{Ra}}}_{3{\text{MH}}}~(\mathrm{\upmu mol}/{\text{d}})}{3.8742~(\mathrm{\upmu mol}/\text{g protein})}$$

**Table 4:** Dynamics of 3-[methyl-^2^H_3_]histidine during peak lactation (d 14 to 18) for sows fed control (CON; 18.44% CP; *n* = 4) and Optimal (OPT; 14.00% CP + CAA; *n* = 4) diets^1^

Item	Diet		SEM^2^	*P*-value
	CON	OPT		
No. of sows	4	4		
Rate of appearance (Ra)^3^, μmol/h	203.57	154.90	22.55	0.178
Area under the curve (AUC)^4^, h	0.938	1.235	0.146	0.200
Half life^5^, h	32.77	31.82	1.88	0.732

^1^Data are least squares means

^2^Maximum value of the standard error of the means

^3^ $${\text{Ra}}=\frac{\text{Bolus dose}}{{\text{AUC}}}$$

^4^ $${\text{AUC}}=\sum_{{\text{i}}=1}^{{\text{n}}}\frac{{{\text{A}}}_{{\text{i}}}}{{{\text{k}}}_{{\text{i}}}}$$

^5^ $$\text{Half life}=\frac{{\text{Ln}}(2)}{{{\text{k}}}_{{\text{n}}}}$$

#### Efficiency of lysine for lactation

Lysine utilization efficiency for lactation was calculated as follows (Eq. 16):

16 $$\text{Efficiency of Lys }(\mathrm{\%})=\frac{\text{Protein net synthesis }({\text{mmol}}/{\text{h}})}{{{\text{Ra}}}_{{\text{Lys}}}\left({\text{mmol}}/{\text{h}}\right)-\text{Lys oxidation}\ ({\text{mmol}}/{\text{h}})}=\frac{\text{Protein synthesis }\left({\text{mmol}}/{\text{h}}\right)-\text{protein breakdown}\ ({\text{mmol}}/{\text{h}})}{{{\text{Ra}}}_{{\text{Lys}}}\left({\text{mmol}}/{\text{h}}\right)-\text{Lys oxidation}\ ({\text{mmol}}/{\text{h}})}\times 100$$

### Statistical analysis

Data were confirmed for homogeneity of residual variance and normality of residuals by Mixed Procedures and Univariate Procedures of SAS 9.4 (SAS Inst. Inc., Cary, NC, USA) before ANOVA analysis (Mixed model procedures).

For the lysine balance and protein kinetic estimation, two sows from CON were removed from the data set. In one case, both ear vein catheters lost patency at the time of infusion, and in the other case, one of the 2 ear vein catheters lost patency. The latter sow, however, was used for the estimation of muscle protein breakdown, which only required one catheter. Therefore, the number of sows for the lysine balance data and protein kinetic estimation were 5 and 3 for OPT and CON, respectively.

For the analysis of lysine enrichment in plasma and milk, the following model was used:

$$\text{Enrichment of lysine}\ =\ \text{diet} + \text{hour}+ block + sow + \text{diet} \times \text{hour} + e$$

The Enrichment of lysine depended on the fixed effects of diet **(**OPT vs. CON**)**, and sampling hour, with hour included as repeated measurement. The random effects included *block* and individual *sow*. The interactive effect of diet × hour was also included*.*

For the analysis of lysine balance, body protein breakdown and synthesis, and muscle protein breakdown rate, identified as “Response”, the following model was used:

$$\mathrm{Response }=\mathrm{ diet }+ block + sow + e$$

The Response depended on the fixed effects of diet **(**OPT vs. CON**)**. The random effects included *block* and individual *sow*.

Differences between treatments were declared at *P* < 0.05 and tendencies at *P* ≤ 0.1.

## Results

### Lactation performance

Lactation performance during the 21-d period and milk yield and nutrient concentrations between d 14 and 18 are presented in Table 5. Sow initial BW and ADFI did not differ between OPT and CON diets. Litter growth rate of sows fed OPT diet was greater than those fed CON diet (*P* < 0.05).

**Table 5:** Lactation performance of all sows fed control (CON; 18.44 % CP) and Optimal (OPT; 14.00% CP + CAA) diets over a 21-d lactation period^1^

Item	Diet		SEM^2^	*P*-value
	CON	OPT		
No. of sows	8	9		
Parity	3.5	3.3	0.3	0.781
Sow ADFI, kg/d	5.84	5.81	0.03	0.676
Sow initial BW, kg	258	242	6.0	0.193
Sow BW change, kg	5.5^*^	-1.7	2.4	0.129
Sow initial back fat, mm	19.9	19.3	1.1	0.770
Sow back fat change, mm	−0.1	−1.4^*^	0.7	0.341
Litter size^3^				
d 1	10.1	10.3	0.3	0.721
d 21	9.4	10.1	0.2	0.121
Litter growth rate (d 1–21), kg/d	2.27	2.64	0.12	0.048
Piglet ADG (d 1–21), g/d	249	263	11	0.356
Litter growth rate (d 14–18), kg/d	2.60	2.99	0.26	0.223
Piglet ADG (d 14–18), g/d	282	298	30	0.659
Milk yield (d 14–18)^4^, kg/d	11.79	13.28	0.87	0.172
Milk protein yield (d 14–18)^5^, g/d	608.60	685.32	44.91	0.172

^1^Data are least squares means

^2^Maximum value of the standard error of the means

^3^Litter size after standardization (within 24 h after parturition)

^4^Estimated according to Theil et al. [15]

^5^Milk yield × 5.16% protein in milk [6]

^*^Body weight and back fat change differed from 0 (*P* = 0.037 and *P* = 0.048, respectively)

### Lysine balance and efficiency of utilization

Lysine balance values are presented in Table 3. The SID Lys intake, Lys oxidation, flux and Lys associated with protein synthesis did not differ between OPT and CON diets (Table 3). Compared to sows fed CON, those fed OPT had greater efficiency of Lys (0.62 vs. 0.50; *P* < 0.05) and tended to have a lower (*P* = 0.069) released Lys associated with protein breakdown.

### Whole body protein synthesis, whole body protein breakdown and fractional muscle protein breakdown

Whole body protein breakdown rate and synthesis rate tended to be lower (*P* = 0.069 and *P* = 0.109, respectively) and protein turnover efficiency (synthesis: breakdown) tended to be greater (*P* = 0.060) in sows fed OPT compared to those fed CON (Table 6). Whole body protein net synthesis (i.e., whole body protein synthesis − whole body protein breakdown) did not differ between OPT and CON diets.

**Table 6:** Body protein synthesis and breakdown of sows fed control (CON; 18.44% CP) and Optimal (OPT; 14.00% CP + CAA) diets during peak lactation (d 14 to 18)^1^

Item	Diet		SEM^2^	*P*-value
	CON	OPT		
No. of sows	3	5		
Whole body protein breakdown, g/d	1,051.46	649.75	114.80	0.069
Muscle protein breakdown^3^, g/d	1261.07	959.57	278.04	0.178
Fractional muscle protein breakdown^4^, %/d	5.59	4.84	0.73	0.197
Whole body protein synthesis, g/d	2,093.19	1,672.65	141.90	0.109
Whole body protein net synthesis^5^, g/d	1,041.72	1,022.90	32.45	0.741
Protein synthesis/ protein breakdown	2.02	2.65	0.166	0.060

^1^Data are least squares means

^2^Maximum value of the standard error of the means

^3^ $$\text{Muscle protein breakdown }({\text{g}}/{\text{d}})=\frac{{{\text{Ra}}}_{3{\text{MH}}}\ (\mathrm{\upmu mol}/{\text{d}})}{3.8742\ (\mathrm{\upmu mol}/\text{g protein})}$$

^4^ $\text{Fractional muscle protein breakdown }(\mathrm{\%}/{\text{d}})=\frac{{{\text{Ra}}}_{3{\text{MH}}}\ (\mathrm{\upmu mol}/{\text{d}})\times 100}{\text{total protein bound }3\text{MH pool }(\mathrm{\upmu mol})}$ , where total protein bound 3MH pool = muscle protein mass (g) × 3.8742 μmol 3MH/g protein and muscle protein mass = 8.21% × sow BW (kg) [20]

^5^Whole body protein net synthesis = whole body protein synthesis − whole body protein breakdown

For estimation of muscle protein breakdown rate, an additional sow in OPT treatment lost patency of both catheters, therefore the number of sows was 4 in each of the treatment. A 3-exponential model best fitted the 3MH decaying curve (Fig. 1) and pseudo-*R*^2^ were > 0.995. Muscle protein breakdown rate and fractional muscle protein breakdown rate (%) did not differ (*P* = 0.197) between sows fed OPT and CON diets (4.84% and 5.59%, respectively) (Table 6).

### Enrichment of lysine

Lysine enrichment in plasma (panel a) and milk (panel b) is presented in Fig. 3. The lysine enrichment in plasma did not differ between diets and time. Lysine enrichment in milk was lower (*P* < 0.01) in sows fed OPT compared to sows fed CON diets and differed over time (*P* < 0.01). There was no interaction between diets and time.

### fDynamics of 3-[methyl-^2^H_3_]histidine

Plasma isotopic enrichment of 3-MH following 3-MH bolus infusion is presented in Fig. 1, and relevant dynamic parameters are presented in Table 4. Plasma isotopic enrichment of 3-MH of sows fed CON was lower (*P* < 0.001) than that for OPT diet. Time effects of 3-MH were significant (*P* < 0.001) in both treatments of CON and OPT, and no interaction effect between diet and time (*P* = 0.894) was detected.

## Discussion

Previous studies showed that lactating sows fed low CP diets with CAA supplementation had greater milk casein yield [2, 3], and utilization efficiency of N and EAA [4, 5]. The improvement of milk yield however was at the expense of sow BW and maternal N retention [3, 5]. Zhang et al. [8] suggested that feeding diets with improved AA balance triggered nutrient repartitioning to milk at the expense of maternal adipose tissue rather than protein tissue. Maternal body fat loss affects subsequent reproductive performance and compromises the overall production efficiency during the sow’s life span [21]. Therefore, commercial implementation of diets with aggressing reduction in CP with CAA supplementation to achieve improved AA balance will not only depend on their impact on lactation performance and production efficiency but also on ensuring that long-term maternal body protein and lipid reserves are not compromised.

The mechanisms behind the reduced maternal N retention in sows fed improved AA balance diets reported in earlier studies [4, 5] are unclear. Reduced maternal body protein synthesis, greater body protein breakdown, or a combination of thereof can dictate maternal N balance during lactation. In this study however, BW and backfat change during lactation did not differ between OPT and CON sows. Of note, sows fed OPT had no change in BW with a small loss in backfat while sows fed CON gained 5.5 kg with no change in backfat. Body protein kinetics in this study (Table 6) dictated that whole body protein net synthesis (whole body protein synthesis − whole body protein breakdown) of sows fed CON and OPT were close (1,041.72 vs. 1,022.90 g/d), but note that whole body protein net synthesis of lactating sows included milk protein yield and maternal protein deposition. Milk protein yield was greater in OPT than CON as mirrored by litter growth rate (Table 5). Consequently, maternal protein deposition was greater in CON than OPT which aligns with the observation that body weight increased in sows fed CON while there was no change of body weight in sows fed OPT (Table 5). In addition, increased milk production in sows fed OPT suggest that OPT diet led sows to partition more dietary nutrient towards milk than maternal reserves, in other words, sows fed OPT were more motivated to produce milk even at the expense of maternal deposition.

**Fig. 4:**
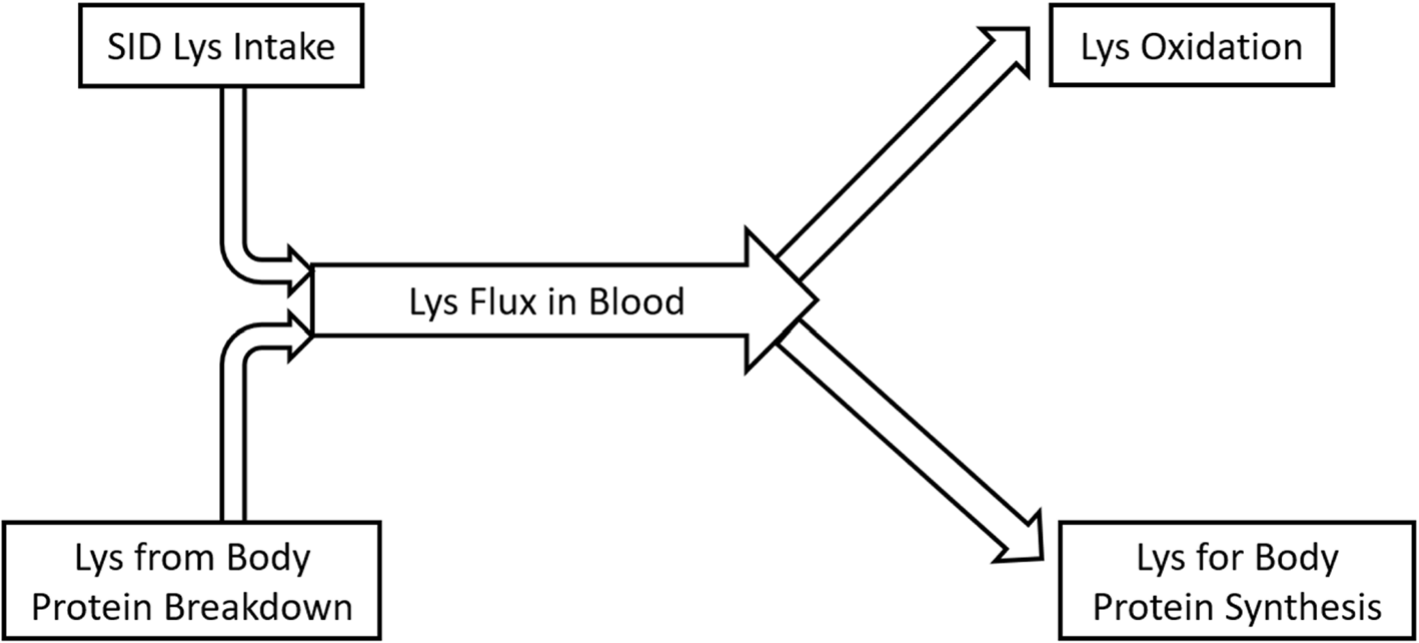
Diagram of lysine balance in lactating sows at fed state

**Fig. 5:**
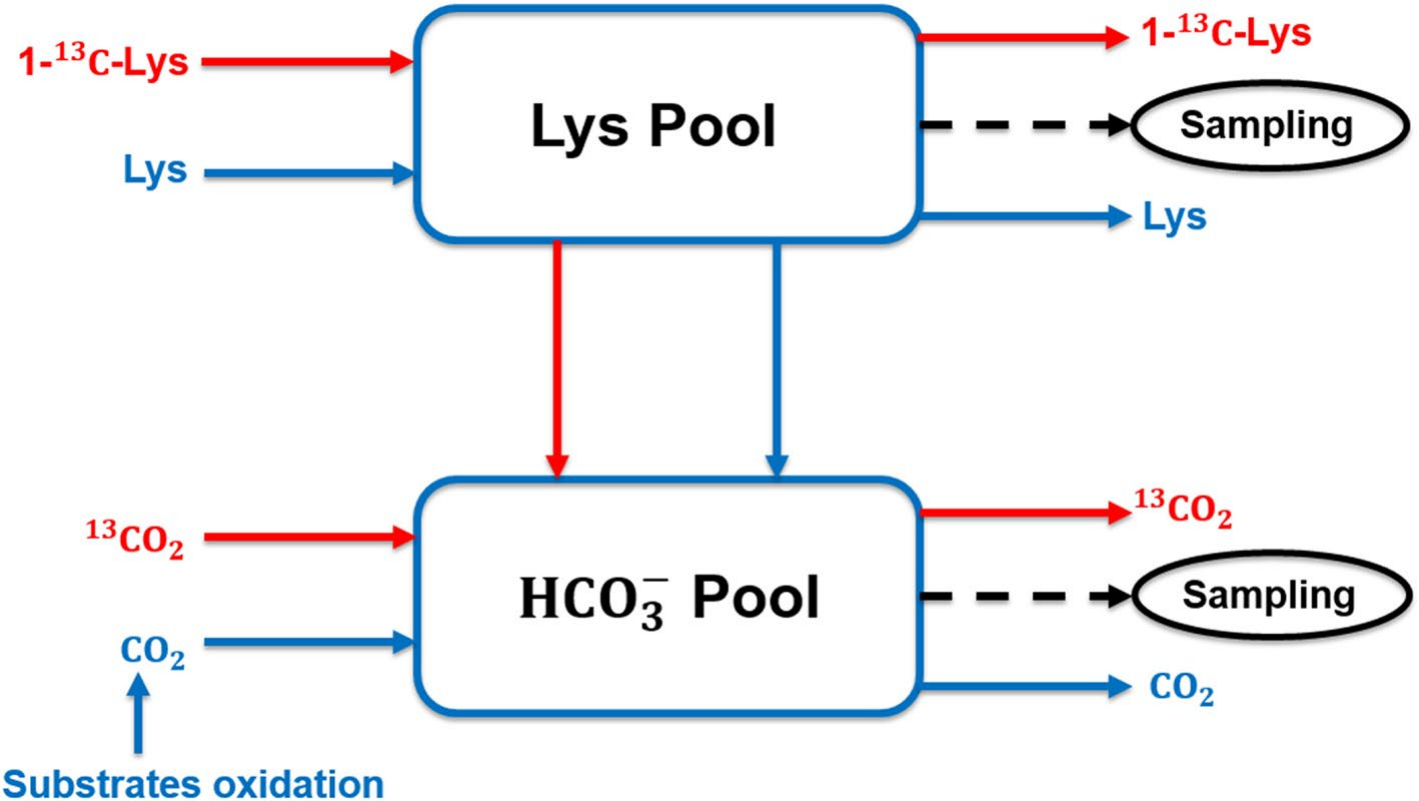
Representation of a two-pool model to estimate lysine oxidation

This study used Lys as representative AA of body protein to analyze whole body protein turnover. In essence, Lys flux in the blood was contributed by dietary Lys intake and Lys released by body protein breakdown, and free Lys in the blood could be directed to either Lys oxidation or Lys incorporation into body protein (Fig. 4). Thus, body protein breakdown and synthesis could be estimated by measuring Lys flux in blood and Lys oxidation. The carbon dioxide released by Lys oxidation remains in the blood bicarbonate pool and mixed with carbon dioxide from other substrate oxidation (Fig. 5). By priming the bicarbonate pool, the baseline production rate of carbon dioxide can be estimated based on bicarbonate enrichment and constant infusion rate of labeled bicarbonate during prime-constant infusion of bicarbonate (Eq. 1). The release of labeled carbon dioxide due to labeled Lys oxidation was proportional to the baseline production rate of carbon dioxide according to enrichment of bicarbonate during prime-constant infusion of Lys (Eqs. 2 and 3).

Milk protein synthesis represents the difference between whole body protein synthesis and breakdown, assuming that maternal protein retention is close to zero, since maternal tissue mobilization is majorly comprised of body fat rather than body protein [5, 8]. According to this assumption, milk protein output rate measured by isotopic technique (Eq. 8) was 1,023 to 1,042 g/d, which aligns well with a previous study where 957 g/d milk protein synthesis was reported using a N balance approach [5]. When compared to traditional method where milk protein synthesis is the product of milk yield and milk protein concentration (645 to 675 g/d; Eq. 9), the isotopic-predicted milk protein synthesis (1,023 to 1,042 g/d) appears overestimated. Guan et al. [7] reported milk protein synthesis of 575 g/d as the net balance between sow whole mammary protein synthesis (975 g/d) and breakdown rate (400 g/d), corroborating the values reported here (645–675 g/d) using the traditional method. Nitrogen balance techniques tend to overestimate actual nitrogen retention [22, 23], as observed herein with the isotope technique (Eq. 8). Note that the estimated muscle protein breakdown rate according to 3MH method in this study was 960 to 1,261 g/d (Eq. 15), which was greater than the protein breakdown rate (650 to 1,051 g/d; Eq. 8) based on the Lys flux. On the other hand, milk protein synthesis rate per metabolic BW (BW^0.75^) were 10.25 and 9.85 g/d/kg^0.75^ for OPT and CON, respectively in this study, supporting a previously reported value of 11.57 g/d/kg^0.75^ (using Val as representative AA) [24]. Thus, overestimation of milk protein synthesis (Eq. 8) was majorly attributed to an underestimation of protein breakdown rather than overestimation of protein synthesis. The underestimation of body protein breakdown according to Lys flux (Eq. 5) may be partially due to the tendency to overestimate feed intake [23], although feed waste was minimized in this study. Nevertheless, it is also important to note that estimated muscle protein breakdown (15.4 to 17.8 μmol/kg/d; Eq. 15; Table 6) and fractional breakdown (4.84–5.59%/d; Eq. 14; Table 6) in this study was greater than those reported for lactating gilt (3.4%/d, 12.0 μmol/kg/d) using the same 3MH method [17]. It is speculated that the multiparous lactating sow may mobilize body protein more readily compared to the lactating gilt.

In this study, milk protein yield of lactating sows fed OPT diet did not differ from those fed control diet neither when an isotopic method nor the traditional method were used. Although there was no difference between whole body protein synthesis and breakdown, the absolute values of protein synthesis and breakdown were both lower in sows fed OPT diet compared to CON diet, suggesting less whole-body protein turnover in sows fed the OPT diet. In support of this view, previous studies also showed a decreased protein breakdown rate reflected by lower urea nitrogen output when sows were fed reduced protein diets [3, 5]. The biological process of protein turnover is energetically costly [1, 25]. Zhang et al. [5] reported feeding sows with an improved AA balance diet was associated with higher energy efficiency, lending support to the current observation.

The Lys efficiency for sows fed different levels of dietary protein based on the NRC [6] approach was previously determined [4, 5], with greater efficiency values (0.68 and 0.66, respectively) found during peak lactation (d 14–18) in sows fed low CP diets balanced with CAA. Herein, greater Lys utilization efficiency values, determined using a different approach, were also found in sows fed OPT (0.62) compared to CON (0.50). The estimation of Lys utilization efficiency was based on Lys balance parameters, i.e., Lys flux in blood, SID Lys intake and Lys oxidation (Fig. 4), and the assumption that net protein synthesis (protein synthesis − protein breakdown) represents milk protein synthesis, with negligible maternal body retention. The true Lys utilization efficiency is the ratio between “Lys in milk” and “Lys for milk”, thus Lys utilized for maintenance should be excluded in the denominator [6] as follows:

$$\text{Lys utization efficiency}=\frac{\text{Lys output in milk }\left({\text{g}}/{\text{d}}\right)-\text{Lys mobilized from body protein }({\text{g}}/{\text{d}}) }{\text{SID Lys intake }\left({\text{g}}/{\text{d}}\right)-\text{Lys for maintenance}({\text{g}}/{\text{d}})}$$

In this study, the whole-body Lys flux was corrected by excluding Lys oxidation (Eq. 16), which corresponds to the Lys requirement for maintenance. Guan et al. [7] reported that Lys flux partitioned to the mammary glands as percentage of whole-body Lys flux was 56% in sows fed a conventional diet, which is comparable to the Lys efficiency values of 50% to 62% observed in this study.

## Conclusion

Feeding lactation sows with an improved AA balance diet did not affect milk protein yield and reduced whole-body protein turnover. The reduced whole-body protein turnover resulted from a decrease in both whole-body protein synthesis and breakdown rate, with a tendency for greater protein synthesis to protein breakdown ratio (2.65 vs. 2.02).

Efficiency of Lys was also greater during peak lactation, together suggesting higher efficiency of energy use. These results imply that the lower maternal N retention observed in lactating sows fed improved AA balance diets in previous studies may be a result of greater partitioning of AA towards milk rather than greater body protein breakdown.

## Data Availability

All data generated or analyzed during this study are available from the corresponding author on request.

## Abbreviations

AA: Amino acid
ADG: Average daily gain
AUC: Area under the curve
BW: Body weight
CAA: Crystalline amino acid
CP: Crude protein
EAA: Essential amino acid
SEM: Standard error of the mean
SID: Standardized ileal digestibility
3-MH: 3-[methyl-^2^H_3_]histidine

## References

[1] Zhang S, Trottier NL. Dietary protein reduction improves the energetic and amino acid efficiency in lactating sows. Anim Prod Sci. 2019;59:1980–90.

[2] Chamberlin DP. Impacts of reducing dietary crude protein with crystalline amino acid supplementation on lactating sow performance, nitrogen utilization and heat production. East Lansing: MS Thesis, Michigan State University; 2017.

[3] Huber L, de Lange CFM, Krogh U, Chamberlin D, Trottier NL. Impact of feeding reduced crude protein diets to lactating sows on nitrogen utilization. J Anim Sci. 2015;93:5254–64. 26641045 10.2527/jas.2015-9382

[4] Huber L, de Lange CFM, Ernst CW, Krogh U, Trottier NL. Impact of improving dietary amino acid balance for lactating sows on efficiency of dietary amino acid utilization and transcript abundance of genes encoding lysine transporters in mammary tissue. J Anim Sci. 2016;94:4654–65. 27898953 10.2527/jas.2016-0697

[5] Zhang S, Qiao M, Trottier NL. Feeding a reduced protein diet with a near ideal amino acid profile improves amino acid efficiency and nitrogen utilization for milk production in sows. J Anim Sci. 2019;97:3882–97. 31394569 10.1093/jas/skz220 PMC6735961

[6] NRC. Nutrient requirements of swine. 11th ed. Washington, DC: National Academy Press; 2012.

[7] Guan X, Bequette BJ, Calder G, Ku PK, Ames KN, Trottier NL. Amino acid availability affects amino acid flux and protein metabolism in the porcine mammary gland. J Nutr. 2002;132:1224–34. 12042438 10.1093/jn/132.6.1224

[8] Zhang S, Johnson JS, Qiao M, Trottier NL. Reduced protein diet with near ideal amino acid profile improves energy efficiency and mitigate heat production associated with lactation in sows. J Anim Sci Biotechno. 2020;11:4. 10.1186/s40104-019-0414-x PMC7006149 32047629

[9] Quesnel H. Nutritional and lactational effects on follicular development in the pig. In: Rodriguez Martinez H, Vallet JL, Ziecik AJ, editors. Control of pig reproduction VIII. Nottingham: Notthingham University Press; 2009. p. 121–34. 19848276

[10] Wientjes JGM, Soede NM, van den Brand H, Kemp B. Nutritionally induced relationships between insulin levels during the weaning-to-ovulation interval and reproductive characteristics in multiparous sows: I. Luteinizing hormone, follicle development, oestrus and ovulation. Reprod Domest Anim. 2012;47:53–61. 21599762 10.1111/j.1439-0531.2011.01801.x

[11] Wientjes JGM, Soede NM, van den Brand H, Kemp B. Nutritionally induced relationships between insulin levels during the weaning-to-ovulation interval and reproductive characteristics in multiparous sows: II. Luteal development, progesterone and conceptus development and uniformity. Reprod Domest Anim. 2012;47:62–8. 21599763 10.1111/j.1439-0531.2011.01802.x

[12] Wientjes JGM, Soede NM, Knol EF, van den Brand H, Kemp B. Piglet birth weight and litter uniformity: effects of weaning-to-pregnancy interval and body condition changes in sows of different parities and crossbred lines. J Anim Sci. 2013;91(5):2099–107. 23463562 10.2527/jas.2012-5659

[13] Kim IY, Schutzler S, Schrader A, Spencer H, Kortebein P, Deutz NEP, et al. Quantity of dietary protein intake, but not pattern of intake, affects net protein balance primarily through differences in protein synthesis in older adults. Am J Physiol-Endoc M. 2015;308(1):E21. 10.1152/ajpendo.00382.2014 PMC4280213 25352437

[14] Claydon AJ, Thom MD, Hurst JL, Beynon RJ. Protein turnover: measurement of proteome dynamics by whole animal metabolic labelling with stable isotope labelled amino acids. Proteomics. 2012;12(8):1194–206. 22577021 10.1002/pmic.201100556

[15] Theil PK, Nielsen TT, Kristensen NB, Labouriau R, Danielsen V, Lauridsen C, et al. Estimation of milk production in lactating sows by determination of deuterated water turnover in three piglets per litter. Acta Agric Scand. 2002;52:221–32.

[16] Rathmacher JA, Link GA, Nissen SL. Measuring of 3-methylhistidine production in lambs by using compartmental-kinetic analysis. Br J Nutr. 1993;69:1. 10.1079/bjn19930075 8329350

[17] Trottier NL. Protein metabolism in the lactating sow. Urbana-Champaign: PhD Dissertation, University of Illinois; 1995.

[18] Marini JC. Quantitative analysis of ^15^N-labeled positional isomers of glutamine and citrulline via electrospray ionization tandem mass spectrometry of their dansyl derivatives. Rapid Commun Mass Spectrom. 2011;25(9):1291–6. 10.1002/rcm.5007 21491530

[19] Verbruggen S, Sy J, Gordon WE, Hsu J, Wu M, Chacko S, et al. Ontogeny of methionine utilization and splanchnic uptake in critically ill children. Am J Physiol Endocrinol Metab. 2009;297(5):E1046–55. 19724018 10.1152/ajpendo.00396.2009 PMC2781350

[20] Rathmacher JA, Nissen SL, Paxton RE, Anderson DB. Estimation of 3-methylhistidine production in pigs by compartmental analysis. J Anim Sci. 1996;74(1):46–56. 8778111 10.2527/1996.74146x

[21] De Rensis F, Gherpelli M, Superchi P, Kirkwood RN. Relationships between backfat depth and plasma leptin during lactation and sow reproductive performance after weaning. Anim Reprod Sci. 2005;90(1–2):95–100. 16257599 10.1016/j.anireprosci.2005.01.017

[22] Kopple JD. Uses and limitations of the balance technique. JPEN J Parenter Enteral Nutr. 1987;11(5 Suppl):79S–85S. 3312697 10.1177/014860718701100511

[23] Spanghero M, Kowalski ZM. Updating analysis of nitrogen balance experiments in dairy cows. J Dairy Sci. 2021;104(7):7725–37. 33838892 10.3168/jds.2020-19656

[24] Hanigan MD, France J, Mabjeesh SJ, McNabb WC, Bequette BJ. High rates of mammary tissue protein turnover in lactating goats are energetically costly. J Nutr. 2009;139(6):1118–27. 19403714 10.3945/jn.108.103002

[25] Wolfe RR. Radioactive and stable isotope tracers in biomedicine: principles and practice of kinetic analysis. Somerset, NJ: John Wiley & Sons Inc; 1992.

